# Persistent Antinuclear Antibodies Positivity Predicts Progression to Systemic Lupus Erythematosus in Paediatric Autoimmune Cytopenias: A Retrospective Cohort Study

**DOI:** 10.31138/mjr.021025.epo

**Published:** 2026-06-01

**Authors:** Valentina Diamantidou, Athanasios Tragiannidis, Maria Trachana, Assimina Galli-Tsinopoulou, Marina-Kely Economou

**Affiliations:** 1 Program of Postgraduate Studies “Adolescent Medicine and Adolescent Health Care”, School of Medicine, Faculty of Health Sciences, Aristotle University of Thessaloniki, Thessaloniki, Greece;; 2 1^st^ Department of Paediatrics, School of Medicine, Faculty of Health Sciences, Aristotle University of Thessaloniki, Thessaloniki, Greece;; 3 2^nd^ Department of Paediatrics, School of Medicine, Faculty of Health Sciences, AHEPA University General Hospital, Aristotle University of Thessaloniki, Thessaloniki, Greece

**Keywords:** adolescent, antibodies, antinuclear, cytopenias, lupus erythematosus, systemic, thrombocytopenia

## Abstract

**Objective:**

Autoimmune cytopenias (AIC) may be associated with positive antinuclear antibodies (ANA) and can precede the onset of systemic lupus erythematosus (SLE). This study assessed ANA as a predictive factor for progression to SLE in adolescents with AIC.

**Methods:**

Medical records from a paediatric haematology referral centre were retrospectively reviewed. Presence of ANA (titer>1:80) was recorded in 90 adolescents and children with AIC. Possible progression to SLE was evaluated in the group.

**Results:**

A total of 90 patients with AIC were included, with a median age of 9 years and a predominance of ITP (67/90). ANA positivity was identified in 35/90 patients with AIC, most frequently in females (25/35). Among ANA-positive patients, 6/35 eventually developed SLE, whereas none of the ANA-negative patients progressed to SLE. Of the patients who developed SLE, the majority were female (5/6), with 5/6 initially diagnosed with autoimmune thrombocytopenia and 1 with Evans syndrome. The median age at AIC onset for those who later developed SLE was 10.4 years, and the median time interval between AIC diagnosis and SLE onset was 2.5 years. Additionally, 10/35 ANA-positive patients met high-risk criteria for future SLE according to Lambers et al. (2020), despite not fulfilling EULAR/ACR-2019 criteria for SLE at the time of review.

**Conclusions:**

Persistent ANA positivity in patients with AIC strongly predicts subsequent SLE, particularly in females older than 10 years with ITP. This finding warrants further investigation in larger, prospective studies, including follow-up of patients transitioning to adult care, as SLE may manifest later in adulthood.

## INTRODUCTION

Childhood-onset systemic lupus erythematosus (cSLE) is a rare autoimmune disease characterised by a wide spectrum of clinical and biological manifestations.^1^ The clinical spectrum of cSLE is often non-specific, which may delay diagnosis and complicate early clinical risk assessment.^2,3^ The incidence of cSLE ranges from 3.3 to 8.8 cases per 100,000 children, accounting for approximately 10%–20% of all cases of systemic lupus erythematosus (SLE).^1^ The most common age of onset for cSLE is adolescence, with an average age of onset between 11 and 12 years.^4,5^ cSLE is more frequently observed in non-Caucasian populations.^6–8^ The overall female-to-male ratio is estimated at 5.4:1, which increases with age (pre-pubertal = 3.3:1; peri-pubertal = 5.24:1; adolescent = 7.25:1).^6,9^ Patients with SLE experience higher mortality rates than the general population, with these rates being particularly elevated among youth.^10^ Based on the latest updated 2019 SLE classification criteria, a required finding for diagnosis is the presence of positive antinuclear antibodies with a titre of ≥1:80, while the remaining criteria are categorised into seven clinical fields (that include haematological findings) and three immunological fields, with scores ranging from 2 to 10. Patients presenting with a score of ≥ 10 are classified as having a SLE diagnosis.^11^ In 2020, Lambers et al. proposed the definition of incomplete systemic lupus (iSLE) so to identify patients who exhibit signs of lupus but do not meet the diagnostic classification criteria.^12^ This definition intended to include patients at high risk for developing SLE or experiencing severe organ involvement.^12^ ANA-related AIC falls within this category.

Haematological manifestations [including autoimmune thrombocytopenia (ITP), autoimmune haemolytic anaemia (AIHA), autoimmune neutropenia, and Evans syndrome (ES)] in systemic lupus erythematosus (SLE) are present in up to 75% of patients at the onset of cSLE.^13,14^ Among these, autoimmune cytopenias (AIC) affect approximately one-third of SLE patients, and their presence is associated with a more severe disease course.^14,15^ Haematological abnormalities, particularly ITP and AIHA, may precede the diagnosis of SLE by several years and represent an early autoimmune phenotype.^16^ Additionally, haematological disorders are more prevalent in children than in adults, their onset sometimes preceding the clinical manifestation of SLE by several years.^14^

The present study aims at retrospectively evaluating the presence of ANA in adolescents with AIC as a predictive factor for progression to SLE.

## PATIENTS AND METHODS

### Study material

The study material was drawn from the outpatient records of a Paediatric Haematology referral centre. The studied period was September 1996 through October 2023. A retrospective review of these records was conducted. For the purposes of this study, the population included all adolescents and/or pre-pubertal patients with AIC whose follow-up continued into adolescence. Only patients who underwent testing for antinuclear antibodies (ANA) at both the initial diagnosis of AIC and during follow-up were included. The study population consisted of 90 patients, who formed the study group. The study size was determined by including all eligible patients seen at our centre during the study period. Patients were excluded if they had been diagnosed with systemic lupus erythematosus (SLE) before the onset of AIC, had AIC of secondary aetiology at initial diagnosis, were in an acute infection phase, were on long-term medication that could cause false-positive ANA results, or had only been followed up before puberty. Autoimmune neutropenia was excluded because, unlike ITP, AIHA, and ES, current evidence does not clearly support an increased risk of systemic lupus erythematosus in children with isolated autoimmune neutropenia. In our centre, ANA testing is not routinely performed for isolated neutropenia, and these patients do not undergo standardised long-term follow-up comparable to that of patients with other AIC subtypes. Including autoimmune neutropenia would therefore introduce clinical heterogeneity and limit the comparability of our results.

Patients were categorised based on the type of AIC (ITP, AIHA, or ES), and the presence of antinuclear antibodies was recorded using the indirect immunofluorescence method (cut-off value ≥1:80, based on the EULAR/ACR-2019 criteria), both at the time of AIC diagnosis and during follow-up.^11^

ANA testing was performed using indirect immunofluorescence (IIF) on HEp-2 cells throughout the entire study period. Although minor updates in commercial kits occurred as part of routine laboratory quality improvement, the diagnostic platform (IIF), cut-off titre (≥1:80), and interpretation criteria remained unchanged. All samples were processed in the same institutional immunology laboratory, ensuring comparability of results over time.

Data from the patient medical history were collected. Specifically, demographic characteristics (age and gender), comorbidities, and family history (presence of autoimmune disease in first- and second-degree relatives) were recorded. Additionally, duration of follow-up and re-evaluation at the Paediatric Haematology outpatient clinic were documented, as well as the treatments received (corticosteroids, IVIG, immunosuppressive drugs, splenectomy, thrombopoietin receptor agonists, monoclonal antibodies and/or blood transfusions) during follow-up.

Finally, clinical and laboratory parameters at diagnosis and during follow-up were recorded according to the SLE diagnostic criteria issued by the European Alliance of Associations for Rheumatology (EULAR) and the American College of Rheumatology (ACR) in 2019. Regarding laboratory findings, complete blood count and full biochemical analysis at the time of the patient presentation at the Paediatric Haematology Outpatient Clinic were documented. Additionally, the presence of immunological criteria, i.e., as antiphospholipid antibodies (anticardiolipin, anti-β2GPI, lupus anticoagulant), complement proteins (C3, C4), and specific SLE-related antibodies (anti-dsDNA or anti-Sm) monitored throughout the follow-up period. Other nuclear antigen antibodies such as anti-Ro/SSA, anti-La/SSB, and anti-RNP were not systematically tested throughout the study period and therefore could not be reliably included in the analysis. Only SLE-specific antibodies routinely performed in all patients (anti-dsDNA and anti-Sm) were included in the dataset.

This retrospective observational study was reported in accordance with the STROBE (Strengthening the Reporting of Observational Studies in Epidemiology) guidelines. Given the retrospective design, potential sources of bias include incomplete documentation and variability in follow-up assessments. To minimize bias, all available medical records were systematically reviewed using predefined criteria.

### Statistical Analysis

To summarise the data, absolute and relative frequencies (%), measures of central tendency (mean and median values), and measures of variability (minimum and maximum values, standard deviations) were calculated. The Mann-Whitney test was used to compare groups of children concerning quantitative parameters. The chi-square (χ^2^) test was applied to compare groups of children regarding categorical/qualitative variables. In the Mann-Whitney and chi-square (χ^2^) tests, the observed significance level (p-values) was calculated using the Monte Carlo simulation method.^17^ This method ensures reliable inferential conclusions, even when the methodological assumptions of the tests (large samples, random samples, independent measurements, symmetric distributions, absence of extreme outliers) are not met. Statistical analyses were conducted using the SPSS software package, version 27.0 (IBM Inc., Chicago, IL, USA). In all statistical tests, the significance level was predetermined at α = 0.05 (*p* ≤ 0.05). Missing data were handled using complete-case analysis.

## RESULTS

During the study period, 90 patients (50/90 female) with AIC, with a median age of 9 years (range: 1–16.5 years), were followed at the Paediatric Haematology Unit of the First Department of Paediatrics of Aristotle University of Thessaloniki. The majority of patients were diagnosed with ITP (67/90), followed by ES (16/90) and AIHA (7/90). The median follow-up duration was four years (1–15 years). Among these 90 patients, 9 had additional autoimmune diseases including 5 cases of Hashimoto’s disease, 2 cases of type 1 diabetes mellitus, 1 case of Raynaud’s syndrome and 1 case of Crohn’s disease, Additionally, 16/90 of patients had a first- or second-degree relative with a reported autoimmune disease in their family history. The most common autoimmune diseases, in order of frequency, were hypothyroidism, rheumatoid arthritis, inflammatory bowel disease, type 1 diabetes mellitus, connective tissue disorders, and Sjögren’s syndrome. During the follow-up period, 64/90 of patients with AIC required treatment.

### ANA-positive patients

During follow-up**,** 35/90 of patients were found to be positive for antinuclear antibodies (ANA) at some point, as determined by indirect immunofluorescence (cut-off value ≥1:80). The vast majority of these patients were female (25/35). Of the 35 patients with positive ANA, 25 had ITP, 7 had ES, and only 3 were diagnosed with AIHA. Among patients with positive ANA, more than half (19/35) tested positive at the time of their haematological disease diagnosis, while 11/35 of patients developed ANA positivity within the next three years. Additionally, ANA remained positive throughout follow-up in 18/35 of patients, while 17/35 showed periodic ANA positivity.

Regarding the medical history of patients with AIC and positive ANA, 6/35 of patients had a coexisting autoimmune disease, and 11/35 had a first- or second-degree relative with a diagnosed autoimmune disease. Finally, 24/35 of patients required treatment for the underlying haematological disorder during follow-up. **Table 1** summarises the characteristics of patients with ANA positive AIC.

**Table 1.** Characteristics of 35 patients with ANA positive AIC.

Patients	ANA(+) n=35	ANA(+) SLE (+) n=6	ANA(+) SLE (−) n=29

Sex ratio (female/male)	25/10	5/1	20/9

Age (in years) of AIC diagnosis			
Median (range)	8.5	10	8.2

AIC type	ITP: 25/35	ITP: 5/6	ITP: 20/29
	ES: 7/35	ES: 1/6	ES:6/29
	AIHA: 3/35	AIHA: 0/6	AIHA:3/29

Autoimmune disease	6/35	2/6	4/29

First- or second-degree relative with autoimmune disease	11/35	2/6	8/29

Median follow-up after AIC diagnosis (in years)	5	2.5	5

Treatment	24/35	4/6	20/29

AIC: autoimmune cytopenias; AIHA: autoimmune haemolytic anaemia; ANA: antinuclear antibodies; ES: Evans syndrome; ITP: autoimmune thrombocytopenia; SLE: systemic lupus erythematosus.

### Patients with SLE

At the end of the study, 6 of 35 patients with positive ANA were diagnosed with SLE according to the 2019 EULAR/ACR criteria. Among these patients, five had ITP and one had ES. Five of these patients were girls. All patients with SLE were persistently ANA positive until the diagnostic criteria for SLE were met. No patient with negative ANA developed SLE. Five of six patients were ANA positive at the time of AIC diagnosis, whereas one became ANA positive one year later. The time to SLE diagnosis varied among patients. In the first patient, SLE was diagnosed almost immediately following AIC diagnosis. The second patient was diagnosed with SLE one year after the diagnosis of AIC, the third patient after two years, the fourth after three years, the fifth after four years, and the sixth patient after seven years of AIC diagnosis. The median time to diagnosis of SLE was 2.5 years following AIC diagnosis. It is worth noting that 2/6 patients had an underlying autoimmune disease (two Hashimoto’s disease), and 2/6 had first- or second-degree relatives with autoimmune rheumatic diseases (SLE and Rheumatoid arthritis). Finally, 4/6 of patients received treatment for their hematologic disorder, in addition to treatment for cSLE by the end of the study. Table 1 presents the characteristics of the patients with SLE.

### High risk patients for developing SLE

Based on the review by Lambers et al. (2020), the classification of “incomplete” SLE has been proposed, addressing patients that may exhibit some signs of SLE but not yet meeting the disease classification criteria.^12^ These are patients considered to be at high risk for developing SLE in the future. In the current study, 10/35 of ANA positive AIC patients met the classification criteria proposed by Lambers et al. (2020).^12^ The characteristics of high-risk patients are detailed in **Table 2**.

**Table 2.** Characteristics of patients at high risk for developing SLE.

1^st^ Patient	ANA(+), thrombocytopenia and positive family history of systemic autoimmune disease (Rheumatoid arthritis)
2^nd^ Patient	ANA(+), thrombocytopenia and positive family history of systemic autoimmune disease (Sjögren syndrome, SLE)
3^rd^ Patient	ANA(+), thrombocytopenia and positive family history of systemic autoimmune disease (psoriatic arthritis)
4^th^ Patient	ANA(+), thrombocytopenia and positive family history of systemic autoimmune disease (SLE)
5^th^ Patient	ANA(+), thrombocytopenia and positive family history of systemic autoimmune disease (psoriatic arthritis)
6^th^ Patient	ANA(+), thrombocytopenia and low complement value
7^th^ Patient	ANA(+), thrombocytopenia and low complement value
8^th^ Patient	ANA(+), thrombocytopenia and low complement value
9^th^ Patient	ANA(+), thrombocytopenia and low complement value
10^th^ Patient	ANA(+), thrombocytopenia and Anti-Sm (+)

ANA: antinuclear antibodies; Anti-Sm: Anti-Smith antibodies; SLE: systemic lupus erythematosus.

## DISCUSSION

This retrospective study evaluated the association between ANA positivity and the risk of developing SLE in adolescents with AIC. Among patients with AIC, 35/90 tested positive for ANA (titre ≥1/80) at some point during follow-up. Of these, 6/35 developed SLE, representing 6.66% of all AIC cases. The observed progression of ANA-positive AIC to SLE in our cohort is comparable to that reported in recent large multicentre paediatric studies.^16^ None of the patients with negative ANA developed SLE, and all those who eventually developed SLE remained ANA-positive throughout follow-up. The vast majority of those who developed SLE were girls (5 of 6) and had ITP (5 of 6). The median age of patients with AIC who were later diagnosed with SLE was 10.4 years, while the median time interval between AIC and SLE diagnosis was 2.5 years.

In the present study, 35/90 of patients with AIC were ANA(+) at some point during follow-up (ANA titre ≥1/80). In similar studies investigating the co-existence of AIC and other autoimmune diseases, lower ANA positivity rates have been reported.^16,18–20^ These differences may arise from variations in inclusion criteria, particularly with regards to patient age, given that the present study exclusively included adolescents or due to differences in follow-up duration.

Regarding the rates of SLE development in paediatric patients with AIC and ANA positivity, the findings of the present study are in accordance to a previous report.^16^ This cohort study assessed the risk of developing SLE in 1,803 children (<18 years old) with AIC. According to this study, 6/35 of patients with AIC and ANA positivity developed SLE at a median age of 14.5, whereas none of the ANA(−) patients did. In our smaller, adolescence focused study, 6/35 of patients with AIC and ANA(+) developed SLE, whereas no ANA(−) patients did.

In our study group, the majority of patients with AIC had ITP (5/6), with only one diagnosed with ES. Although our group was small, the finding is consistent with that of previous reports, also showing ITP is the most common type of AIC in patients who eventually develop SLE, with a reported incidence that reaches 62%.^8,16,21–23^ However, it remains unclear whether paediatric patients with ITP have an inherently higher risk of developing SLE compared to those with other types of AIC or whether this trend simply reflects the higher overall incidence of ITP among AIC.

The overall reported rate of children with AIC developing SLE in the present study (6.6%) is in accordance with other studies.^16,24^ Lower rates have been observed in adult patients with ITP (1.79%–4.7%) or AIHA (0.82%),^25,26^ although the duration of follow-up in different studies varies.

Regarding patient sex, in the present report the majority of patients with ANA- positive AIC were girls (25/35), as were those who later developed SLE (5/6). A recent study reported similar results, with girls comprising 71% (252/355) of patients with ANA- positive AIC and an impressive 91.1% (72/79) of those ultimately developed SLE. Girls with ITP appear to be at a higher risk of developing SLE, as supported by two other retrospective studies: Hazzan et al. (2006) examined risk factors for future development of SLE in children with ITP and concluded that older girls with high ANA titres (≥1/40) were at higher risk.^19^ Similarly, Kittivisuit et al. (2021) found that development of SLE in girls with ITP was statistically more common than in boys.^27^ These findings suggest that female sex may be an independent risk factor for development of SLE in children with AIC. In a retrospective study of 2022, the median time to diagnosis of SLE among 50 patients initially presented with ITP was 2.9 years.^28^ In a recent study by Granel et al. (2024), most patients developed SLE within 3.4 years after their AIC diagnosis.^16^ In the present study, the median time between AIC and SLE diagnosis was 2.5 years. However, the lack of follow-up beyond adulthood excludes cases that may develop SLE later in life. It is worth noting that 10 patients in our study did not meet the EULAR/ACR-2019 criteria for the diagnosis of SLE. However, based on a study by Lambers et al. (2020), these cases could be classified as high-risk for future development of SLE.^12^ All these patients fall into the category proposed by the authors, that includes ANA≥1:80 and at least two of the following criteria: (1) haematological manifestations (haemolytic anaemia, leukopenia or lymphopenia <1,000/mm^3^ at least once, or thrombocytopenia <100,000/mm^3^ at least once), (2) immunological features (anti-dsDNA, anti-Sm, antiphospholipid antibodies, low complement, or direct Coombs test), and (3) a positive family history of autoimmune rheumatic diseases (first- or second-degree relatives with an autoimmune rheumatic disease). Some combinations proposed by the authors have already been classified as SLE according to the EULAR/ACR-2019 criteria, while the rest, although not recommended by an expert panel, indicate a subgroup of patients who would likely benefit from closer monitoring. In all patients considered as high-risk according to the above criteria, the development of SLE reaches a 55% rate.^12^ Similar observations have been reported in longitudinal studies of incomplete systemic lupus erythematosus, where patients with persistent ANA positivity and limited clinical criteria may progress to classified SLE over time, supporting the need for close clinical monitoring of this subgroup.^12,29^

In our study, the median follow-up time from AIC diagnosis was five years, with six patients ultimately developing SLE. Two of these patients were initially classified as high risk according to the criteria of Lambers et al. (2020) due to a positive family history of systemic autoimmune diseases.^12^ It is possible that the remaining 10 high-risk patients, although not meeting the official SLE criteria at study follow up, may develop SLE in the future. This hypothesis is supported by the fact that four of these 10 patients had a score of 8 according to the EULAR/ACR-2019 criteria, whereas one had a score of 9. Therefore, paediatric patients with AIC, positive ANA, and immunologic features or a positive family history of systemic autoimmune disease may require closer monitoring. Whether these patients could benefit from early treatment for SLE is also an issue that needs to be addressed.

The findings of this study add to the growing body of evidence that autoimmune cytopenias may represent an early manifestation of systemic autoimmunity in children and adolescents. While previous studies have examined ANA positivity in paediatric ITP or AIHA, relatively few have assessed its predictive value across a broader AIC population with extended follow-up into adolescence. Our study supports earlier observations that ANA positivity is strongly associated with subsequent SLE development and extends current knowledge by demonstrating that persistent ANA positivity was present in all patients who developed SLE, whereas none of the ANA-negative patients did. This finding highlights the importance of persistent, rather than transient, ANA positivity when assessing future risk of systemic autoimmune disease in children with AIC. Furthermore, by identifying a substantial subgroup of ANA-positive AIC patients who meet criteria for incomplete SLE, our data may help clinicians identify children who require closer surveillance.

This study also provides long-term data from a Greek paediatric cohort, a population for which evidence on autoimmune cytopenias and their evolution into systemic autoimmune disease remains limited. Most published cohorts originate from North America, Northern Europe, or Asia, and geographic or ethnic differences may influence disease presentation and timing of SLE onset. By contributing data from a Mediterranean setting, our study enhances the geographic diversity of available evidence and demonstrates that the predictive value of ANA positivity in AIC appears consistent across different populations. These findings may support the development of regionally adapted monitoring strategies for ANA-positive cytopenias within the Greek healthcare system.

This study has some limitations. In terms of study type, it is a single-centre, retrospective, observational study, so the level of scientific evidence is relatively limited. Moreover, the study excluded patients with autoimmune neutropenia, as the risk of developing SLE was not assessed. Finally, although the follow-up of the patients was long-term, it was not extended into adulthood, potentially leading to an underestimation of the incidence of SLE. However, the overall sample was adequate in terms of focusing on the adolescent subgroup. The absence of systematic testing for anti-Ro, anti-La, and anti-RNP antibodies limited our ability to evaluate their potential predictive value, and therefore these markers were not included in the analysis.

## CONCLUSIONS

In children with AIC, persistent ANA(+) titres ≥1/80, appear to be a predictor of progression to SLE, especially in girls over 10 years of age. Given that early diagnosis of SLE is of utmost importance for timely intervention, ANA testing, a low-cost and widely available test, is a valuable tool that should not be overlooked. It is also important to establish ongoing collaboration between paediatric and adult centres, which will extend well after the transition, in order to identify the true incidence of SLE diagnosis in children with AIC onset.

## OPEN DATA SHARING POLICY

The raw datasets generated and analysed during the current study are available from the corresponding author upon reasonable request.

## DATA CONFIDENTIALITY

All data were fully anonymised prior to analysis to protect patient confidentiality.

## NOTE

An earlier version of this work was presented as an e-poster at the 10th Panhellenic congress of Paediatric Haematology and Oncology, 21–23 November 2024, Athens, Greece.
